# What Can We Do to Improve Child Health in Southern Italy?

**DOI:** 10.1371/journal.pmed.0020250

**Published:** 2005-08-23

**Authors:** Maurizio Bonati, Rita Campi

## Abstract

Southern Italy has one of the highest rates of poverty in Europe, and children's health status in this region is alarming. Bonati and Campi outline the crucial policies that would address this crisis.

Two-thirds of all poor Italian families are in the south. Of the 20.7 million people (36.1% of Italians) living in southern Italy, 7.3 million (35.4%) are poor, living on less than €521 per month. Some 4.6 million of these people (63.3%) are extremely poor, living on less than €435 per month [1,2]. With such an economic profile, if southern Italy was thought of as an independent European country, it would be the European country with the highest poverty rate, weighted for national income. In this article, we discuss health inequalities in Italy, with a particular focus on the very poor health status of children in southern Italy, and we ponder possible health policies to address these inequalities [3].

How does poverty in Italy compare with poverty in the rest of Europe? Within the 25 European Union countries, in 2001 about 68 million people lived on less than 60% of their respective national median income [4]. Of these 68 million, 3.6 million were children under five years old [4]. The proportion of people at risk of poverty, redistributed based on a value of one as the EU average, ranges from a minimum of 0.3 in Slovakia and a maximum of 1.4 in Ireland. Italy's average value is 0.6, but is 0.4 in the north, 0.9 in the center, and 2.3 in the south.

Southern Italy can, therefore, be considered the European country with the highest risk of poverty. The proportion of people who are under five years old in southern Italy is 6.4%—thus, southern Italy has the third highest percentage of children under five, after Ireland and Cyprus.

## The Crisis of Poor Child Health in Southern Italy

The monitoring and planning of child welfare is crucial for the health of a community and for a nation's public health in general. Social and economic factors are determinants of child health inequalities [5,6]. Inequalities within countries, including developed ones, are well-known to affect the health of populations, in particular that of minorities such as children [7,8].

Two-thirds of cases accounting for the national infant mortality rate (3.3 per 1,000 live births) involve neonatal death, especially within the first week of life (early neonatal mortality). Neonatal mortality varies widely between the different Italian regions, with rates four times higher in the south (Sicily and Basilicata's rates are 5.7 per 1,000 live births) than in the north (Friuli Venezia Giulia's rate is 1.3 per 1,000 live births) (Figure 1 shows the different regions of Italy) [9].

**Figure 1:**
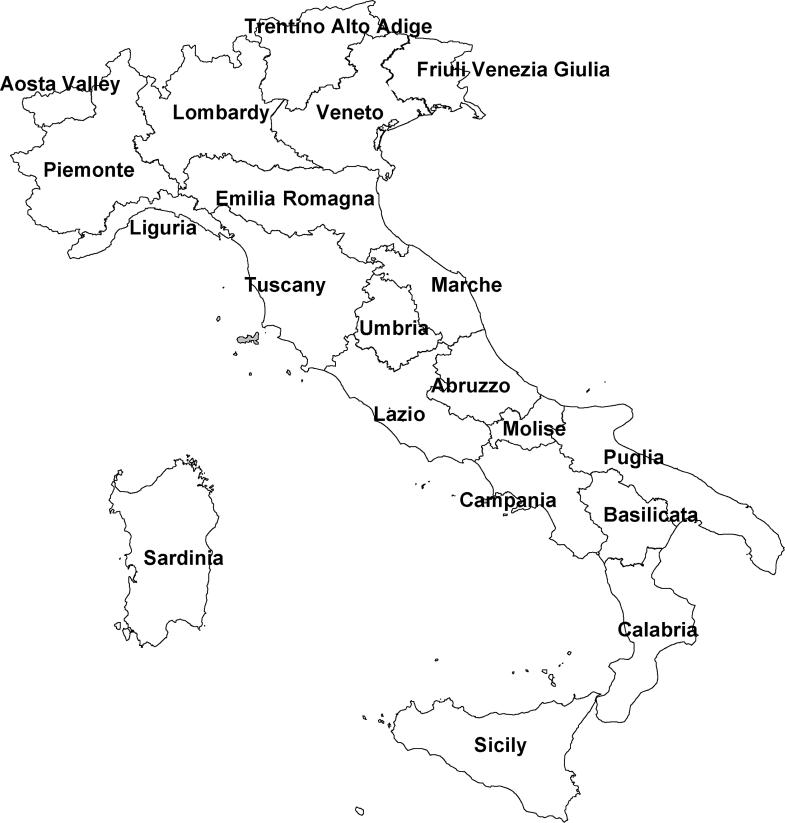
The Different Regions of Italy

Although birth weight is one of the recognized factors that contribute to infant mortality, there are no regional variations in the distribution of low and very low birth weight infants. However, the risk of early neonatal death for low birth weight infants born, for example, in Sicily or Abruzzo is more than nine times higher than the risk for newborns in the Aosta Valley (rates of 91.7 and 101.7 per 1,000 live births versus 11.4 per 1,000 live births, respectively). One possible explanation for this could be a wide gap in perinatal care quality (health-care structures and quality of health assistance) associated with latitude in Italy [10].

Hospitalization rates during childhood (age 14 years or younger) are similar between regions (average 151.8 per 1,000 inhabitants), and are higher in infants, in male children, and for respiratory system diseases. However, more than 22% of hospitalized children from the Basilicata and Molise regions and more than 13% of hospitalized children from the Calabria and Abruzzo regions are treated in hospitals in northern or central Italy, also suggesting a lack of adequate pediatric services in the south, both in terms of quality and quantity [9]. Because of unequal distribution of services in the south, in many circumstances people would need to move across the region to access health services, and traveling to the north may be a simpler option since, according to cultural beliefs, the quality of public services are thought to be better in the north [11].

**The Italian National Health Service**

The Italian National Health Service (NHS), introduced in 1978, provides universal coverage and comprehensive health care, free of cost or at a nominal charge upon delivery. The NHS is structured in three different levels of public authority: the central government, 20 regions, and 196 local health units covering an average of 290,000 persons each. Despite a strong, recent devolution policy, shifting power to the regions, the Italian government provides most of the funding for the NHS and is responsible for ensuring the NHS provides uniform, essential levels of health-care services across the country.

One of the most significant features of the public system is the gate-keeping function of the family practitioner (or family pediatrician for children under six years). Every Italian resident is required to register with a family practitioner, who is responsible for prescribing pharmaceuticals and diagnostic procedures and for referring patients to specialists and hospitals.

Although the World Health Report 2000 ranked the Italian health-care system second among 191 countries (France was the first) with respect to health status, fairness in financial contribution, and responsiveness to people's expectations of a health-care system [35], the dissatisfaction of Italians with respect to the efficiency and quality of their NHS is the highest in Europe [36]. Moreover, there is considerable disparity between the southern and northern Italian regions, with respect to satisfaction with NHS performance: for example, 19% in Sicily versus 53% in Emilia Romagna [37]. Tight budgets and the need to restrain rising health-care expenditures have led the NHS to undertake several cost-containment measures to encourage cost-conscious behavior by consumers and providers, accentuating economic and social interregional disparities [38].

The inequalities across Italy in children's health status are not only related to inequalities in treatment, but also in health prevention. The vaccine uptake rate for measles by a child's second birthday, for example, ranges from 54.9% in Calabria to 89.6% in Tuscany [12]. Italy continues to have the lowest coverage rate for measles among European countries; a national campaign was recently launched to increase the coverage to an expected rate of 90%–95% [13].

## Geographical Variations in Social and Educational Opportunities

Regional inequalities in the provision of social and educational services can have a profound effect on the welfare of Italian children. There is evidence to suggest that the south of Italy has inadequate public services for its needs. For example, Campania and Sicily, the regions in the south with the highest birth rates in the country (11.5 and 10.4 births per 1,000 inhabitants, respectively), have the lowest national rate of access to nursery school (2.2% and 4.7%, for 0- to 2-year-old children). Both of these regions, along with Calabria and Puglia (all in southern Italy), show a rate of primary school abandonment (i.e., children abandon school completely) that is 2.5 times higher than in the Friuli Venezia Giulia region (about 24% versus 9%) [14].

There are also regional variations in the youth unemployment rate (i.e., the rate for those aged 15–19 years). The rate ranges from 65.2% and 61.4% for Calabria and Sicily, respectively, to 7.1% for Trentino Alto Adige.

**The Italian Welfare State**

The Italian welfare state is a mix of (1) occupationally fragmented social security systems, especially with regard to pensions (such as the German-style democratic social policy) and (2) social security systems that are based on universal coverage, as in the field of health-care provision (such as the British-style liberal social policy, based on universal social insurance schemes) [39]. Italy, thus, has a mixed model of social and occupational provision. The Italian health-care and social service sectors are, moreover, highly reliant on the support of nonprofit organizations.

The state is also charged with providing financial support for low-income workers and different forms of housing assistance. Unemployment benefits are provided by the government in the form of cash transfers based on contributions. Old-age pension is insurance-based and proportional to contributions. Education for a child age 10–15 years old is free but vocational training for those who are older is not free. The state is responsible for secondary schooling and university education [40]. The rapid increase in the elderly population, with the decrease of the overall adult population that provides health care, shows the growing need for a reorganization of the whole economic and welfare systems [41].

Over the past 6–7 years, Italy has been steadily sliding down the ranking in the United Nations Development Programme's Human Development Index (21st place in 2002), whereas it remains in 11th place in the Human Poverty Index. With the Berlusconi government (the head of which is the richest Italian, who also controls around 75% of the media and 90% of national broadcasting), decades of social achievements have been lost because of structural reforms and privatizations. These reforms benefit only a small proportion of the population, with most Italians seeing a decline in their quality of life, an increase in inequalities, and re-establishment of privileges for the few [42].

Children in southern Italy, therefore, face a constellation of risks—a high rate of school dropout, a low youth employment rate, and a higher likelihood of living in difficult family circumstances (involving factors such as low family educational level, family poverty, and families doing illegal work). Children in the north are much less likely to experience this set of risks [15].

## The Ethics of Child Poverty

Inequalities in society raise fundamental ethical questions. In particular, such inequalities challenge us to take actions to improve health, based upon an ethical framework in which human rights and dignity are taken into consideration [16]. Inequality is based on the deprivation of rights such as education, work, and access to social services; inequality also means loss of human dignity, which is in itself linked to poverty [17]. Well-being is not only affected by money and economic status (i.e., gross domestic product and income), but also by social- and health-related rights and opportunities [18]. Children's rights to health must always be a priority, not only between but also within countries, and not only in developing but also in developed countries, until inequalities are overcome [19].

## Policies to Improve Child Health in Southern Italy

Even though its effects have been questioned by the government, income inequality undoubtedly affects health outcomes, even in Italy, where health care and education are mostly public and free, and the unemployed receive social benefits [20]. The reduction of income inequality not only requires policies promoting economic development but also policies minimizing material deprivation (education, health services, transportation, environmental controls, availability of good quality food, quality of housing, and occupational health regulations). In addition, social inequalities may cause negative feelings (stress, shame, and distrust), which translates into poorer health by way of psycho-neuro-endocrine-immunological mechanisms and stress-induced behaviors such as smoking and overeating [21–23].

Initiatives to meet the identified needs should be carefully planned and overseen to ensure their successful and sustainable implementation. Tackling health inequalities requires a working partnership between government, social administration and organizations, the private sector, and civil society, at the national, regional, and local levels [24]. Equity is a permanent ethical and socioeconomic goal that requires adequate capacity, sound policies for improvement, and enough financial investment to ensure (or at least try to ensure) success. Intention to change is obviously not enough if egalitarian values are not widespread and felt by an entire nation, and if the social, cultural, and political causes of health inequalities are not addressed [25]. If dignity is not central to policy change, and rights are neglected, children will remain one of the most marginalized groups in society [26,27].

What are the particular prerequisites for addressing inequities in children's health? Strong and effective programs for improving child equity must be based on adequate, targeted financial, human, and service resource allocations. A starting point for planning such programs must be the systematic, ongoing evaluation of child health. This evaluation should not only take into consideration national averages, but also analyses stratified by socioeconomic and geographic categories, according to worldwide recognized indicators and methods [5]. Such analyses must be performed, and the data used, by national and regional governments to establish and monitor objectives related to health status and health-service use in disadvantaged population groups.

Another prerequisite is the creation of information, which is a powerful and influential tool and is, therefore, essential for improving accountability in a society. Moreover, reduction of child health inequalities can be pursued by promoting useful and adequate information, aimed not only at policymakers and program managers, but also at regional and local communities [28].

Unfortunately, the latest Italian National Health Programme (2003–2005; http://www.ministerosalute.it/psn/psnHome.jsp) ignores the unacceptably wide regional differences in child health care. There is no mention of improving the availability of health-related structures or the quality of assistance in perinatal care or childhood hospitalization. Similarly, the new public-school reform (http://www.istruzione.it/normativa/2004/schemadec210504.shtml) only mentions general principles concerning rights and duties, and ignores the dramatic school abandonment rate in southern Italy. It is, therefore, apparent that the “business as usual” approach, based on national averages, instead of regional and local needs, and supported by inadequate economic resources, can only increase the inequity gap instead of closing it.

## Conclusion

The European experience of the development, implementation, and evaluation of policies and interventions to reduce health inequalities has been well-described [29–34]. For example, the European Network on Interventions and Policies to Reduce Inequalities in Health has recently analyzed policy developments concerning health inequalities in different European countries between 1990 and 2001 [29]. Compared to the rest of Europe, Italy's approach to ensuring the future health of its children is not encouraging. In a country with an aging population and an increasing gap between regions, the implementation of public-health programs focusing on promoting, monitoring, and improving children's well-being should be taken on as a recognized challenge and should be a political commitment. Furthermore, international and intracountry exchanges focused on child health can help enhance and speed up learning.

## Abbreviation

NHS: Italian National Health Service
